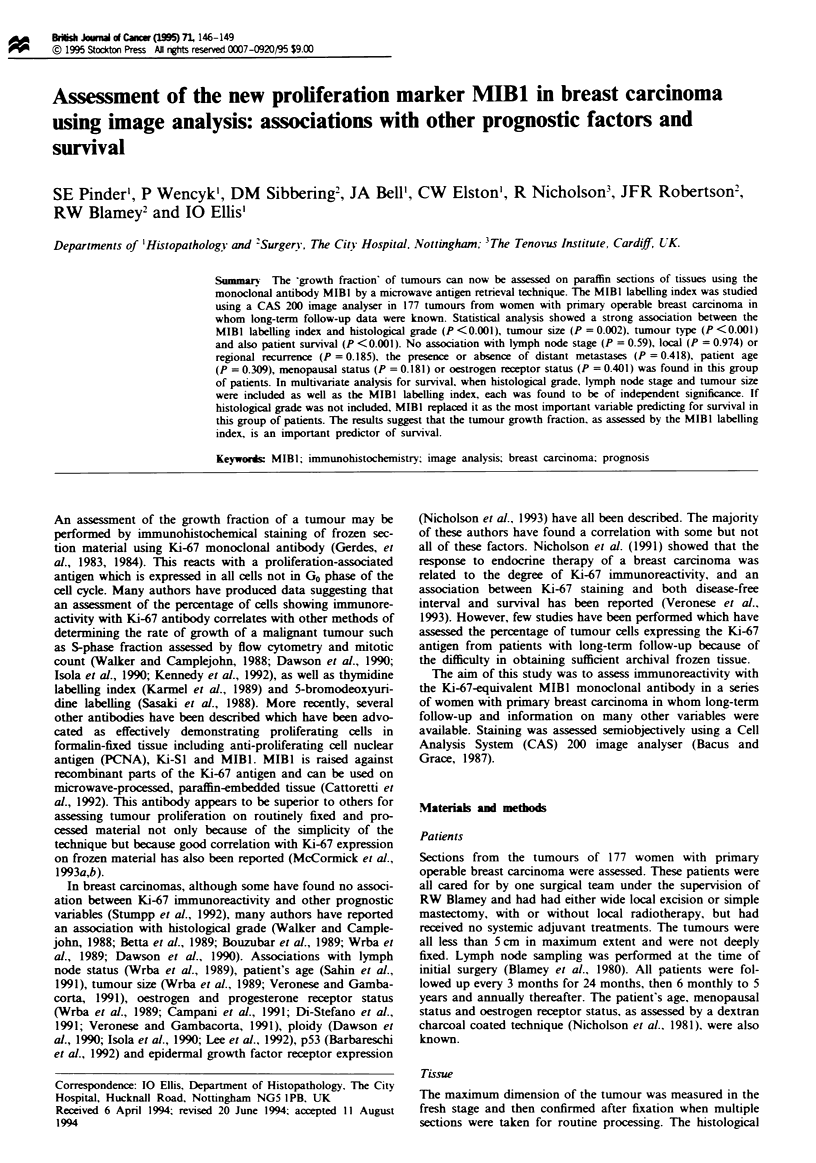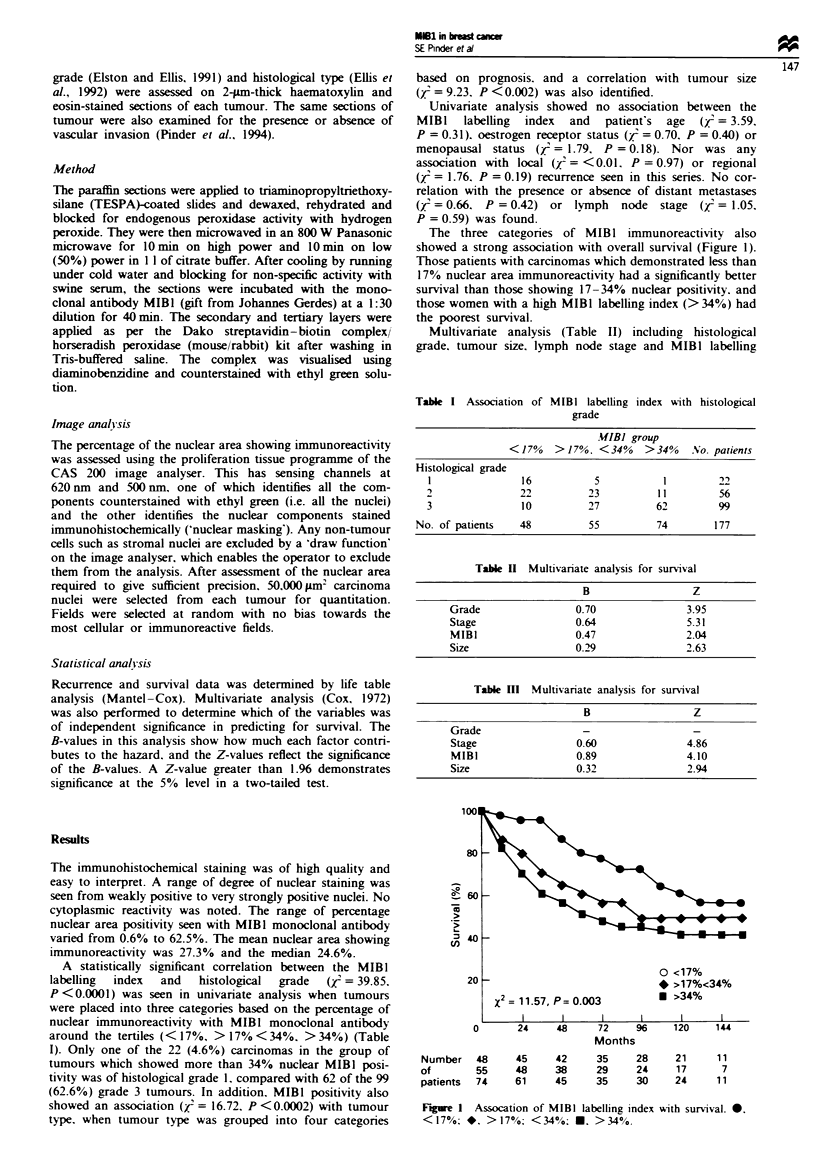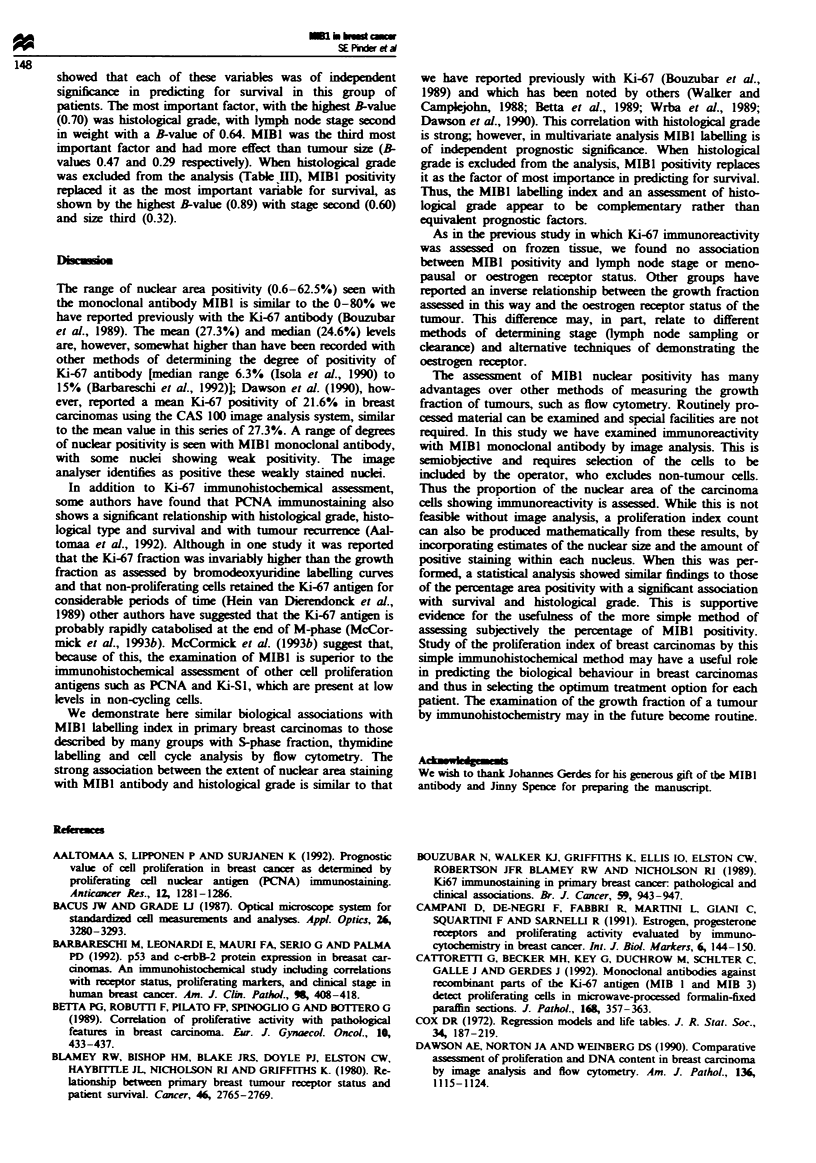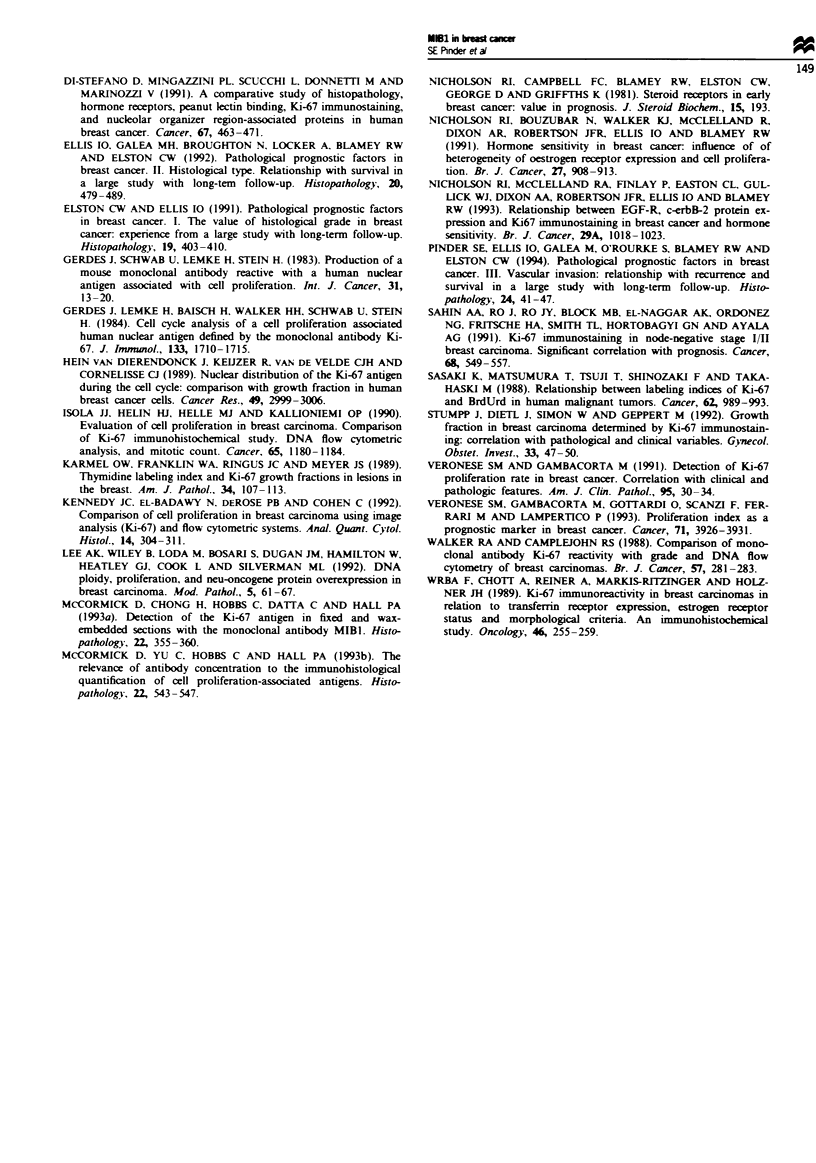# Assessment of the new proliferation marker MIB1 in breast carcinoma using image analysis: associations with other prognostic factors and survival.

**DOI:** 10.1038/bjc.1995.30

**Published:** 1995-01

**Authors:** S. E. Pinder, P. Wencyk, D. M. Sibbering, J. A. Bell, C. W. Elston, R. Nicholson, J. F. Robertson, R. W. Blamey, I. O. Ellis

**Affiliations:** Department of Histopathology, City Hospital, Nottingham, UK.

## Abstract

The 'growth fraction' of tumours can now be assessed on paraffin sections of tissues using the monoclonal antibody MIB1 by a microwave antigen retrieval technique. The MIB1 labelling index was studied using a CAS 200 image analyser in 177 tumours from women with primary operable breast carcinoma in whom long-term follow-up data were known. Statistical analysis showed a strong association between the MIB1 labelling index and histological grade (P < 0.001), tumour size (P = 0.002), tumour type (P < 0.001) and also patient survival (P < 0.001). No association with lymph node stage (P = 0.974) or regional recurrence (P = 0.185), the presence or absence of distant metastases (P = 0.418), patient age (P = 0.309), menopausal status (P = 0.181) or oestrogen receptor status (P = 0.401) was found in this group of patients. In multivariate analysis for survival, when histological grade, lymph node stage and tumour size were included as well as the MIB1 labelling index, each was found to be of independent significance. If histological grade was not included, MIB1 replaced it as the most important variable predicting for survival in this group of patients. The results suggest that the tumour growth fraction, as assessed by the MIB1 labelling index, is an important predictor of survival.


British Journal of Cancer (1995) 71, 146–149

© 1995 Stockton Press All rights reserved 0007-0920/95 $9.00

### Assessment of the new proliferation marker MIB1 in breast carcinoma using image analysis: associations with other prognostic factors and survival

SE Pinder[1], P Wencyk[1], DM Sibbering[2], JA Bell[1], CW Elston[1], R Nicholson[3], JFR Robertson[2], RW Blamey[2] and IO Ellis[1]

*Departments of [1]Histopathology and [2]Surgery, The City Hospital, Nottingham; [3]The Tenovus Institute, Cardiff, UK.*

**Summary** The 'growth fraction' of tumours can now be assessed on paraffin sections of tissues using the monoclonal antibody MIB1 by a microwave antigen retrieval technique. The MIB1 labelling index was studied using a CAS 200 image analyser in 177 tumours from women with primary operable breast carcinoma in whom long-term follow-up data were known. Statistical analysis showed a strong association between the MIB1 labelling index and histological grade ($P<0.001$), tumour size ($P=0.002$), tumour type ($P<0.001$) and also patient survival ($P<0.001$). No association with lymph node stage ($P=0.59$), local ($P=0.974$) or regional recurrence ($P=0.185$), the presence or absence of distant metastases ($P=0.418$), patient age ($P=0.309$), menopausal status ($P=0.181$) or oestrogen receptor status ($P=0.401$) was found in this group of patients. In multivariate analysis for survival, when histological grade, lymph node stage and tumour size were included as well as the MIB1 labelling index, each was found to be of independent significance. If histological grade was not included, MIB1 replaced it as the most important variable predicting for survival in this group of patients. The results suggest that the tumour growth fraction, as assessed by the MIB1 labelling index, is an important predictor of survival.

**Keywords:** MIB1; immunohistochemistry; image analysis; breast carcinoma; prognosis

An assessment of the growth fraction of a tumour may be performed by immunohistochemical staining of frozen section material using Ki-67 monoclonal antibody (Gerdes, *et al.*, 1983, 1984). This reacts with a proliferation-associated antigen which is expressed in all cells not in $G_0$ phase of the cell cycle. Many authors have produced data suggesting that an assessment of the percentage of cells showing immunoreactivity with Ki-67 antibody correlates with other methods of determining the rate of growth of a malignant tumour such as S-phase fraction assessed by flow cytometry and mitotic count (Walker and Camplejohn, 1988; Dawson *et al.*, 1990; Isola *et al.*, 1990; Kennedy *et al.*, 1992), as well as thymidine labelling index (Karmel *et al.*, 1989) and 5-bromodeoxyuridine labelling (Sasaki *et al.*, 1988). More recently, several other antibodies have been described which have been advocated as effectively demonstrating proliferating cells in formalin-fixed tissue including anti-proliferating cell nuclear antigen (PCNA), Ki-S1 and MIB1. MIB1 is raised against recombinant parts of the Ki-67 antigen and can be used on microwave-processed, paraffin-embedded tissue (Cattoretti *et al.*, 1992). This antibody appears to be superior to others for assessing tumour proliferation on routinely fixed and processed material not only because of the simplicity of the technique but because good correlation with Ki-67 expression on frozen material has also been reported (McCormick *et al.*, 1993*a,b*).

In breast carcinomas, although some have found no association between Ki-67 immunoreactivity and other prognostic variables (Stumpp *et al.*, 1992), many authors have reported an association with histological grade (Walker and Camplejohn, 1988; Betta *et al.*, 1989; Bouzubar *et al.*, 1989; Wrba *et al.*, 1989; Dawson *et al.*, 1990). Associations with lymph node status (Wrba *et al.*, 1989), patient's age (Sahin *et al.*, 1991), tumour size (Wrba *et al.*, 1989; Veronese and Gambacorta, 1991), oestrogen and progesterone receptor status (Wrba *et al.*, 1989; Campani *et al.*, 1991; Di-Stefano *et al.*, 1991; Veronese and Gambacorta, 1991), ploidy (Dawson *et al.*, 1990; Isola *et al.*, 1990; Lee *et al.*, 1992), p53 (Barbareschi *et al.*, 1992) and epidermal growth factor receptor expression (Nicholson *et al.*, 1993) have all been described. The majority of these authors have found a correlation with some but not all of these factors. Nicholson *et al.* (1991) showed that the response to endocrine therapy of a breast carcinoma was related to the degree of Ki-67 immunoreactivity, and an association between Ki-67 staining and both disease-free interval and survival has been reported (Veronese *et al.*, 1993). However, few studies have been performed which have assessed the percentage of tumour cells expressing the Ki-67 antigen from patients with long-term follow-up because of the difficulty in obtaining sufficient archival frozen tissue.

The aim of this study was to assess immunoreactivity with the Ki-67-equivalent MIB1 monoclonal antibody in a series of women with primary breast carcinoma in whom long-term follow-up and information on many other variables were available. Staining was assessed semiobjectively using a Cell Analysis System (CAS) 200 image analyser (Bacus and Grace, 1987).

### Materials and methods

### *Patients*

Sections from the tumours of 177 women with primary operable breast carcinoma were assessed. These patients were all cared for by one surgical team under the supervision of RW Blamey and had had either wide local excision or simple mastectomy, with or without local radiotherapy, but had received no systemic adjuvant treatments. The tumours were all less than 5 cm in maximum extent and were not deeply fixed. Lymph node sampling was performed at the time of initial surgery (Blamey *et al.*, 1980). All patients were followed up every 3 months for 24 months, then 6 monthly to 5 years and annually thereafter. The patient's age, menopausal status and oestrogen receptor status, as assessed by a dextran charcoal coated technique (Nicholson *et al.*, 1981), were also known.

### *Tissue*

The maximum dimension of the tumour was measured in the fresh stage and then confirmed after fixation when multiple sections were taken for routine processing. The histological

Correspondence: IO Ellis, Department of Histopathology, The City Hospital, Hucknall Road, Nottingham NG5 1PB, UK
Received 6 April 1994; revised 20 June 1994; accepted 11 August 1994

grade (Elston and Ellis, 1991) and histological type (Ellis *et al.*, 1992) were assessed on 2-μm-thick haematoxylin and eosin-stained sections of each tumour. The same sections of tumour were also examined for the presence or absence of vascular invasion (Pinder *et al.*, 1994).

### *Method*

The paraffin sections were applied to triaminopropyltriethoxysilane (TESPA)-coated slides and dewaxed, rehydrated and blocked for endogenous peroxidase activity with hydrogen peroxide. They were then microwaved in an 800 W Panasonic microwave for 10 min on high power and 10 min on low (50%) power in 1 l of citrate buffer. After cooling by running under cold water and blocking for non-specific activity with swine serum, the sections were incubated with the monoclonal antibody MIB1 (gift from Johannes Gerdes) at a 1:30 dilution for 40 min. The secondary and tertiary layers were applied as per the Dako streptavidin-biotin complex/horseradish peroxidase (mouse/rabbit) kit after washing in Tris-buffered saline. The complex was visualised using diaminobenzidine and counterstained with ethyl green solution.

### *Image analysis*

The percentage of the nuclear area showing immunoreactivity was assessed using the proliferation tissue programme of the CAS 200 image analyser. This has sensing channels at 620 nm and 500 nm, one of which identifies all the components counterstained with ethyl green (i.e. all the nuclei) and the other identifies the nuclear components stained immunohistochemically ('nuclear masking'). Any non-tumour cells such as stromal nuclei are excluded by a 'draw function' on the image analyser, which enables the operator to exclude them from the analysis. After assessment of the nuclear area required to give sufficient precision, 50,000 $\mu m^2$ carcinoma nuclei were selected from each tumour for quantitation. Fields were selected at random with no bias towards the most cellular or immunoreactive fields.

### *Statistical analysis*

Recurrence and survival data was determined by life table analysis (Mantel-Cox). Multivariate analysis (Cox, 1972) was also performed to determine which of the variables was of independent significance in predicting for survival. The *B*-values in this analysis show how much each factor contributes to the hazard, and the *Z*-values reflect the significance of the *B*-values. A *Z*-value greater than 1.96 demonstrates significance at the 5% level in a two-tailed test.

### Results

The immunohistochemical staining was of high quality and easy to interpret. A range of degree of nuclear staining was seen from weakly positive to very strongly positive nuclei. No cytoplasmic reactivity was noted. The range of percentage nuclear area positivity seen with MIB1 monoclonal antibody varied from 0.6% to 62.5%. The mean nuclear area showing immunoreactivity was 27.3% and the median 24.6%.

A statistically significant correlation between the MIB1 labelling index and histological grade ($\chi^2 = 39.85$, $P < 0.0001$) was seen in univariate analysis when tumours were placed into three categories based on the percentage of nuclear immunoreactivity with MIB1 monoclonal antibody around the tertiles (<17%, >17%<34%, >34%) (Table I). Only one of the 22 (4.6%) carcinomas in the group of tumours which showed more than 34% nuclear MIB1 positivity was of histological grade 1, compared with 62 of the 99 (62.6%) grade 3 tumours. In addition, MIB1 positivity also showed an association ($\chi^2 = 16.72$, $P < 0.0002$) with tumour type, when tumour type was grouped into four categories based on prognosis, and a correlation with tumour size ($\chi^2 = 9.23$, $P < 0.002$) was also identified.

Univariate analysis showed no association between the MIB1 labelling index and patient's age ($\chi^2 = 3.59$, $P = 0.31$), oestrogen receptor status ($\chi^2 = 0.70$, $P = 0.40$) or menopausal status ($\chi^2 = 1.79$, $P = 0.18$). Nor was any association with local ($\chi^2 = < 0.01$, $P = 0.97$) or regional ($\chi^2 = 1.76$, $P = 0.19$) recurrence seen in this series. No correlation with the presence or absence of distant metastases ($\chi^2 = 0.66$, $P = 0.42$) or lymph node stage ($\chi^2 = 1.05$, $P = 0.59$) was found.

The three categories of MIB1 immunoreactivity also showed a strong association with overall survival (Figure 1). Those patients with carcinomas which demonstrated less than 17% nuclear area immunoreactivity had a significantly better survival than those showing 17-34% nuclear positivity, and those women with a high MIB1 labelling index (>34%) had the poorest survival.

Multivariate analysis (Table II) including histological grade, tumour size, lymph node stage and MIB1 labelling

**Table I** Association of MIB1 labelling index with histological grade

| | *MIB1 group* <17% | >17%, <34% | >34% | *No. patients* |
|---|---|---|---|---|
| Histological grade | | | | |
| 1 | 16 | 5 | 1 | 22 |
| 2 | 22 | 23 | 11 | 56 |
| 3 | 10 | 27 | 62 | 99 |
| No. of patients | 48 | 55 | 74 | 177 |

**Table II** Multivariate analysis for survival

| | B | Z |
|---|---|---|
| Grade | 0.70 | 3.95 |
| Stage | 0.64 | 5.31 |
| MIB1 | 0.47 | 2.04 |
| Size | 0.29 | 2.63 |

**Table III** Multivariate analysis for survival

| | B | Z |
|---|---|---|
| Grade | – | – |
| Stage | 0.60 | 4.86 |
| MIB1 | 0.89 | 4.10 |
| Size | 0.32 | 2.94 |

**Figure 1** Assocation of MIB1 labelling index with survival. ●, <17%; ◆, >17%; <34%; ■, >34%.

showed that each of these variables was of independent significance in predicting for survival in this group of patients. The most important factor, with the highest *B*-value (0.70) was histological grade, with lymph node stage second in weight with a *B*-value of 0.64. MIB1 was the third most important factor and had more effect than tumour size (*B*-values 0.47 and 0.29 respectively). When histological grade was excluded from the analysis (Table III), MIB1 positivity replaced it as the most important variable for survival, as shown by the highest *B*-value (0.89) with stage second (0.60) and size third (0.32).

### Discussion

The range of nuclear area positivity (0.6–62.5%) seen with the monoclonal antibody MIB1 is similar to the 0–80% we have reported previously with the Ki-67 antibody (Bouzubar *et al.*, 1989). The mean (27.3%) and median (24.6%) levels are, however, somewhat higher than have been recorded with other methods of determining the degree of positivity of Ki-67 antibody [median range 6.3% (Isola *et al.*, 1990) to 15% (Barbareschi *et al.*, 1992)]; Dawson *et al.* (1990), however, reported a mean Ki-67 positivity of 21.6% in breast carcinomas using the CAS 100 image analysis system, similar to the mean value in this series of 27.3%. A range of degrees of nuclear positivity is seen with MIB1 monoclonal antibody, with some nuclei showing weak positivity. The image analyser identifies as positive these weakly stained nuclei.

In addition to Ki-67 immunohistochemical assessment, some authors have found that PCNA immunostaining also shows a significant relationship with histological grade, histological type and survival and with tumour recurrence (Aaltomaa *et al.*, 1992). Although in one study it was reported that the Ki-67 fraction was invariably higher than the growth fraction as assessed by bromodeoxyuridine labelling curves and that non-proliferating cells retained the Ki-67 antigen for considerable periods of time (Hein van Dierendonck *et al.*, 1989) other authors have suggested that the Ki-67 antigen is probably rapidly catabolised at the end of M-phase (McCormick *et al.*, 1993*b*). McCormick *et al.* (1993*b*) suggest that, because of this, the examination of MIB1 is superior to the immunohistochemical assessment of other cell proliferation antigens such as PCNA and Ki-S1, which are present at low levels in non-cycling cells.

We demonstrate here similar biological associations with MIB1 labelling index in primary breast carcinomas to those described by many groups with S-phase fraction, thymidine labelling and cell cycle analysis by flow cytometry. The strong association between the extent of nuclear area staining with MIB1 antibody and histological grade is similar to that we have reported previously with Ki-67 (Bouzubar *et al.*, 1989) and which has been noted by others (Walker and Camplejohn, 1988; Betta *et al.*, 1989; Wrba *et al.*, 1989; Dawson *et al.*, 1990). This correlation with histological grade is strong; however, in multivariate analysis MIB1 labelling is of independent prognostic significance. When histological grade is excluded from the analysis, MIB1 positivity replaces it as the factor of most importance in predicting for survival. Thus, the MIB1 labelling index and an assessment of histological grade appear to be complementary rather than equivalent prognostic factors.

As in the previous study in which Ki-67 immunoreactivity was assessed on frozen tissue, we found no association between MIB1 positivity and lymph node stage or menopausal or oestrogen receptor status. Other groups have reported an inverse relationship between the growth fraction assessed in this way and the oestrogen receptor status of the tumour. This difference may, in part, relate to different methods of determining stage (lymph node sampling or clearance) and alternative techniques of demonstrating the oestrogen receptor.

The assessment of MIB1 nuclear positivity has many advantages over other methods of measuring the growth fraction of tumours, such as flow cytometry. Routinely processed material can be examined and special facilities are not required. In this study we have examined immunoreactivity with MIB1 monoclonal antibody by image analysis. This is semiobjective and requires selection of the cells to be included by the operator, who excludes non-tumour cells. Thus the proportion of the nuclear area of the carcinoma cells showing immunoreactivity is assessed. While this is not feasible without image analysis, a proliferation index count can also be produced mathematically from these results, by incorporating estimates of the nuclear size and the amount of positive staining within each nucleus. When this was performed, a statistical analysis showed similar findings to those of the percentage area positivity with a significant association with survival and histological grade. This is supportive evidence for the usefulness of the more simple method of assessing subjectively the percentage of MIB1 positivity. Study of the proliferation index of breast carcinomas by this simple immunohistochemical method may have a useful role in predicting the biological behaviour in breast carcinomas and thus in selecting the optimum treatment option for each patient. The examination of the growth fraction of a tumour by immunohistochemistry may in the future become routine.

**Acknowledgements**

We wish to thank Johannes Gerdes for his generous gift of the MIB1 antibody and Jinny Spence for preparing the manuscript.

### References

AALTOMAA S, LIPPONEN P AND SURJANEN K (1992). Prognostic value of cell proliferation in breast cancer as determined by proliferating cell nuclear antigen (PCNA) immunostaining. *Anticancer Res.*, **12**, 1281–1286.

BACUS JW AND GRADE LJ (1987). Optical microscope system for standardized cell measurements and analyses. *Appl. Optics*, **26**, 3280–3293.

BARBARESCHI M, LEONARDI E, MAURI FA, SERIO G AND PALMA PD (1992). p53 and c-erbB-2 protein expression in breasat carcinomas. An immunohistochemical study including correlations with receptor status, proliferating markers, and clinical stage in human breast cancer. *Am. J. Clin. Pathol.*, **98**, 408–418.

BETTA PG, ROBUTTI F, PILATO FP, SPINOGLIO G AND BOTTERO G (1989). Correlation of proliferative activity with pathological features in breast carcinoma. *Eur. J. Gynaecol. Oncol.*, **10**, 433–437.

BLAMEY RW, BISHOP HM, BLAKE JRS, DOYLE PJ, ELSTON CW, HAYBITTLE JL, NICHOLSON RI AND GRIFFITHS K. (1980). Relationship between primary breast tumour receptor status and patient survival. *Cancer*, **46**, 2765–2769.

BOUZUBAR N, WALKER KJ, GRIFFITHS K, ELLIS IO, ELSTON CW, ROBERTSON JFR BLAMEY RW AND NICHOLSON RI (1989). Ki67 immunostaining in primary breast cancer: pathological and clinical associations. *Br. J. Cancer*, **59**, 943–947.

CAMPANI D, DE-NEGRI F, FABBRI R, MARTINI L, GIANI C, SQUARTINI F AND SARNELLI R (1991). Estrogen, progesterone receptors and proliferating activity evaluated by immunocytochemistry in breast cancer. *Int. J. Biol. Markers*, **6**, 144–150.

CATTORETTI G, BECKER MH, KEY G, DUCHROW M, SCHLTER C, GALLE J AND GERDES J (1992). Monoclonal antibodies against recombinant parts of the Ki-67 antigen (MIB 1 and MIB 3) detect proliferating cells in microwave-processed formalin-fixed paraffin sections. *J. Pathol.*, **168**, 357–363.

COX DR (1972). Regression models and life tables. *J. R. Stat. Soc.*, **34**, 187–219.

DAWSON AE, NORTON JA AND WEINBERG DS (1990). Comparative assessment of proliferation and DNA content in breast carcinoma by image analysis and flow cytometry. *Am. J. Pathol.*, **136**, 1115–1124.

DI-STEFANO D, MINGAZZINI PL, SCUCCHI L, DONNETTI M AND MARINOZZI V (1991). A comparative study of histopathology, hormone receptors, peanut lectin binding, Ki-67 immunostaining, and nucleolar organizer region-associated proteins in human breast cancer. *Cancer*, **67**, 463-471.

ELLIS IO, GALEA MH, BROUGHTON N, LOCKER A, BLAMEY RW AND ELSTON CW (1992). Pathological prognostic factors in breast cancer. II. Histological type. Relationship with survival in a large study with long-tem follow-up. *Histopathology*, **20**, 479-489.

ELSTON CW AND ELLIS IO (1991). Pathological prognostic factors in breast cancer. I. The value of histological grade in breast cancer: experience from a large study with long-term follow-up. *Histopathology*, **19**, 403-410.

GERDES J, SCHWAB U, LEMKE H, STEIN H. (1983). Production of a mouse monoclonal antibody reactive with a human nuclear antigen associated with cell proliferation. *Int. J. Cancer*, **31**, 13-20.

GERDES J, LEMKE H, BAISCH H, WALKER HH, SCHWAB U, STEIN H. (1984). Cell cycle analysis of a cell proliferation associated human nuclear antigen defined by the monoclonal antibody Ki-67. *J. Immunol.*, **133**, 1710-1715.

HEIN VAN DIERENDONCK J, KEIJZER R, VAN DE VELDE CJH AND CORNELISSE CJ (1989). Nuclear distribution of the Ki-67 antigen during the cell cycle: comparison with growth fraction in human breast cancer cells. *Cancer Res.*, **49**, 2999-3006.

ISOLA JJ, HELIN HJ, HELLE MJ AND KALLIONIEMI OP (1990). Evaluation of cell proliferation in breast carcinoma. Comparison of Ki-67 immunohistochemical study. DNA flow cytometric analysis, and mitotic count. *Cancer*, **65**, 1180-1184.

KARMEL OW, FRANKLIN WA, RINGUS JC AND MEYER JS (1989). Thymidine labeling index and Ki-67 growth fractions in lesions in the breast. *Am. J. Pathol.*, **34**, 107-113.

KENNEDY JC, EL-BADAWY N, DEROSE PB AND COHEN C (1992). Comparison of cell proliferation in breast carcinoma using image analysis (Ki-67) and flow cytometric systems. *Anal. Quant. Cytol. Histol.*, **14**, 304-311.

LEE AK, WILEY B, LODA M, BOSARI S, DUGAN JM, HAMILTON W, HEATLEY GJ, COOK L AND SILVERMAN ML (1992). DNA ploidy, proliferation, and neu-oncogene protein overexpression in breast carcinoma. *Mod. Pathol.*, **5**, 61-67.

MCCORMICK D, CHONG H, HOBBS C, DATTA C AND HALL PA (1993*a*). Detection of the Ki-67 antigen in fixed and wax-embedded sections with the monoclonal antibody MIB1. *Histopathology*, **22**, 355-360.

MCCORMICK D, YU C, HOBBS C AND HALL PA (1993b). The relevance of antibody concentration to the immunohistological quantification of cell proliferation-associated antigens. *Histopathology*, **22**, 543-547.

NICHOLSON RI, CAMPBELL FC, BLAMEY RW, ELSTON CW, GEORGE D AND GRIFFTHS K (1981). Steroid receptors in early breast cancer: value in prognosis. *J. Steroid Biochem.*, **15**, 193.

NICHOLSON RI, BOUZUBAR N, WALKER KJ, MCCLELLAND R, DIXON AR, ROBERTSON JFR, ELLIS IO AND BLAMEY RW (1991). Hormone sensitivity in breast cancer: influence of of heterogeneity of oestrogen receptor expression and cell proliferation. *Br. J. Cancer*, **27**, 908-913.

NICHOLSON RI, MCCLELLAND RA, FINLAY P, EASTON CL, GULLICK WJ, DIXON AA, ROBERTSON JFR, ELLIS IO AND BLAMEY RW (1993). Relationship between EGF-R, c-erbB-2 protein expression and Ki67 immunostaining in breast cancer and hormone sensitivity. *Br. J. Cancer*, **29A**, 1018-1023.

PINDER SE, ELLIS IO, GALEA M, O'ROURKE S, BLAMEY RW AND ELSTON CW (1994). Pathological prognostic factors in breast cancer. III. Vascular invasion: relationship with recurrence and survival in a large study with long-term follow-up. *Histopathology*, **24**, 41-47.

SAHIN AA, RO J, RO JY, BLOCK MB, EL-NAGGAR AK, ORDONEZ NG, FRITSCHE HA, SMITH TL, HORTOBAGYI GN AND AYALA AG (1991). Ki-67 immunostaining in node-negative stage I/II breast carcinoma. Significant correlation with prognosis. *Cancer*, **68**, 549-557.

SASAKI K, MATSUMURA T, TSUJI T, SHINOZAKI F AND TAKAHASKI M (1988). Relationship between labeling indices of Ki-67 and BrdUrd in human malignant tumors. *Cancer*, **62**, 989-993.

STUMPP J, DIETL J, SIMON W AND GEPPERT M (1992). Growth fraction in breast carcinoma determined by Ki-67 immunostaining: correlation with pathological and clinical variables. *Gynecol. Obstet. Invest.*, **33**, 47-50.

VERONESE SM AND GAMBACORTA M (1991). Detection of Ki-67 proliferation rate in breast cancer. Correlation with clinical and pathologic features. *Am. J. Clin. Pathol.*, **95**, 30-34.

VERONESE SM, GAMBACORTA M, GOTTARDI O, SCANZI F, FERRARI M AND LAMPERTICO P (1993). Proliferation index as a prognostic marker in breast cancer. *Cancer*, **71**, 3926-3931.

WALKER RA AND CAMPLEJOHN RS (1988). Comparison of monoclonal antibody Ki-67 reactivity with grade and DNA flow cytometry of breast carcinomas. *Br. J. Cancer*, **57**, 281-283.

WRBA F, CHOTT A, REINER A, MARKIS-RITZINGER AND HOLZNER JH (1989). Ki-67 immunoreactivity in breast carcinomas in relation to transferrin receptor expression, estrogen receptor status and morphological criteria. An immunohistochemical study. *Oncology*, **46**, 255-259.